# Supplementary material for: Are carbon emissions trading and green financial instruments synergistic? -Comprehensive quantitative research based on content analysis
Source: PLoS One. 2024 Mar 7;19(3):e0298601. doi: 10.1371/journal.pone.0298601 (PMC10919601; doi:10.1371/journal.pone.0298601)
Supplement: S1 Data — (ZIP) [file pone.0298601.s001.zip › Data/地级市Z_F工作报告.zip!/─┌├╔╣┼/║⌠║═║╞╠╪╩╨/└┤╫╘╣1⁄2╓┌║┼í╛┬φ┐╦╩2╛▌═°í┐_2018.docx]

各位代表：

现在，我代表国务院，向大会报告过去五年政府工作，对今年工作提出建议，请予审议，并请全国政协委员提出意见。

一、过去五年工作回顾

第十二届全国人民代表大会第一次会议以来的五年，是我国发展进程中极不平凡的五年。面对极其错综复杂的国内外形势，以习近平同志为核心的党中央团结带领全国各族人民砥砺前行，统筹推进“五位一体”总体布局，协调推进“四个全面”战略布局，改革开放和社会主义现代化建设全面开创新局面。党的十九大确立了习近平新时代中国特色社会主义思想的历史地位，制定了决胜全面建成小康社会、夺取新时代中国特色社会主义伟大胜利的宏伟蓝图和行动纲领，具有重大现实意义和深远历史意义。各地区各部门不断增强政治意识、大局意识、核心意识、看齐意识，深入贯彻落实新发展理念，“十二五”规划胜利完成，“十三五”规划顺利实施，经济社会发展取得历史性成就、发生历史性变革。

五年来，经济实力跃上新台阶。国内生产总值从54万亿元增加到82.7万亿元，年均增长7.1%，占世界经济比重从11.4%提高到15%左右，对世界经济增长贡献率超过30%。财政收入从11.7万亿元增加到17.3万亿元。居民消费价格年均上涨1.9%，保持较低水平。城镇新增就业6600万人以上，13亿多人口的大国实现了比较充分就业。

五年来，经济结构出现重大变革。消费贡献率由54.9%提高到58.8%，服务业比重从45.3%上升到51.6%，成为经济增长主动力。高技术制造业年均增长11.7%。粮食生产能力达到1.2万亿斤。城镇化率从52.6%提高到58.5%，8000多万农业转移人口成为城镇居民。

五年来，创新驱动发展成果丰硕。全社会研发投入年均增长11%，规模跃居世界第二位。科技进步贡献率由52.2%提高到57.5%。载人航天、深海探测、量子通信、大飞机等重大创新成果不断涌现。高铁网络、电子商务、移动支付、共享经济等引领世界潮流。“互联网+”广泛融入各行各业。大众创业、万众创新蓬勃发展，日均新设企业由5千多户增加到1万6千多户。快速崛起的新动能，正在重塑经济增长格局、深刻改变生产生活方式，成为中国创新发展的新标志。

五年来，改革开放迈出重大步伐。改革全面发力、多点突破、纵深推进，重要领域和关键环节改革取得突破性进展，主要领域改革主体框架基本确立。简政放权、放管结合、优化服务等改革推动政府职能发生深刻转变，市场活力和社会创造力明显增强。“一带一路”建设成效显著，对外贸易和利用外资结构优化、规模稳居世界前列。

五年来，人民生活持续改善。脱贫攻坚取得决定性进展，贫困人口减少6800多万，易地扶贫搬迁830万人，贫困发生率由10.2%下降到3.1%。居民收入年均增长7.4%、超过经济增速，形成世界上人口最多的中等收入群体。出境旅游人次由8300万增加到1亿3千多万。教育事业全面发展。社会养老保险覆盖9亿多人，基本医疗保险覆盖13.5亿人，织就了世界上最大的社会保障网。人均预期寿命达到76.7岁。棚户区住房改造2600多万套，农村危房改造1700多万户，上亿人喜迁新居。

五年来，生态环境状况逐步好转。制定实施大气、水、土壤污染防治三个“十条”并取得扎实成效。单位国内生产总值能耗、水耗均下降20%以上，主要污染物排放量持续下降，重点城市重污染天数减少一半，森林面积增加1.63亿亩，沙化土地面积年均缩减近2000平方公里，绿色发展呈现可喜局面。

刚刚过去的2017年，经济社会发展主要目标任务全面完成并好于预期。国内生产总值增长6.9%，居民收入增长7.3%，增速均比上年有所加快；城镇新增就业1351万人，失业率为多年来最低；工业增速回升，企业利润增长21%；财政收入增长7.4%，扭转了增速放缓态势；进出口增长14.2%，实际使用外资1363亿美元、创历史新高。经济发展呈现出增长与质量、结构、效益相得益彰的良好局面。这是五年来一系列重大政策效应累积，各方面不懈努力、久久为功的结果。

过去五年取得的全方位、开创性成就，发生的深层次、根本性变革，再次令世界瞩目，全国各族人民倍感振奋和自豪。

五年来，我们认真贯彻以习近平同志为核心的党中央决策部署，主要做了以下工作。

（一）坚持稳中求进工作总基调，着力创新和完善宏观调控，经济运行保持在合理区间、实现稳中向好。这些年，世界经济复苏乏力，国际金融市场跌宕起伏，保护主义明显抬头。我国经济发展中结构性问题和深层次矛盾凸显，经济下行压力持续加大，遇到不少两难多难抉择。面对这种局面，我们保持战略定力，坚持不搞“大水漫灌”式强刺激，而是适应把握引领经济发展新常态，统筹稳增长、促改革、调结构、惠民生、防风险，不断创新和完善宏观调控，确立区间调控的思路和方式，加强定向调控、相机调控、精准调控。明确强调只要经济运行在合理区间，就业增加、收入增长、环境改善，就集中精力促改革、调结构、添动力。采取既利当前更惠长远的举措，着力推进供给侧结构性改革，适度扩大总需求，推动实现更高层次的供需动态平衡。经过艰辛努力，我们顶住了经济下行压力、避免了“硬着陆”，保持了经济中高速增长，促进了结构优化，经济长期向好的基本面不断巩固和发展。

坚持实施积极的财政政策和稳健的货币政策。在财政收支矛盾较大情况下，着眼“放水养鱼”、增强后劲，我国率先大幅减税降费。分步骤全面推开营改增，结束了66年的营业税征收历史，累计减税超过2万亿元，加上采取小微企业税收优惠、清理各种收费等措施，共减轻市场主体负担3万多亿元。加强地方政府债务管理，实施地方政府存量债务置换，降低利息负担1.2万亿元。调整财政支出结构，盘活沉淀资金，保障基本民生和重点项目。财政赤字率一直控制在3%以内。货币政策保持稳健中性，广义货币M2增速呈下降趋势，信贷和社会融资规模适度增长。采取定向降准、专项再贷款等差别化政策，加强对重点领域和薄弱环节支持，小微企业贷款增速高于各项贷款平均增速。改革完善汇率市场化形成机制，保持人民币汇率基本稳定，外汇储备转降为升。妥善应对“钱荒”等金融市场异常波动，规范金融市场秩序，防范化解重点领域风险，守住了不发生系统性风险的底线，维护了国家经济金融安全。

（二）坚持以供给侧结构性改革为主线，着力培育壮大新动能，经济结构加快优化升级。紧紧依靠改革破解经济发展和结构失衡难题，大力发展新兴产业，改造提升传统产业，提高供给体系质量和效率。

扎实推进“三去一降一补”。五年来，在淘汰水泥、平板玻璃等落后产能基础上，以钢铁、煤炭等行业为重点加大去产能力度，中央财政安排1000亿元专项奖补资金予以支持，用于分流职工安置。退出钢铁产能1.7亿吨以上、煤炭产能8亿吨，安置分流职工110多万人。因城施策分类指导，三四线城市商品住宅去库存取得明显成效，热点城市房价涨势得到控制。积极稳妥去杠杆，控制债务规模，增加股权融资，工业企业资产负债率连续下降，宏观杠杆率涨幅明显收窄、总体趋于稳定。多措并举降成本，压减政府性基金项目30%，削减中央政府层面设立的涉企收费项目60%以上，阶段性降低“五险一金”缴费比例，推动降低用能、物流、电信等成本。突出重点加大补短板力度。

加快新旧发展动能接续转换。深入开展“互联网+”行动，实行包容审慎监管，推动大数据、云计算、物联网广泛应用，新兴产业蓬勃发展，传统产业深刻重塑。实施“中国制造2025”，推进工业强基、智能制造、绿色制造等重大工程，先进制造业加快发展。出台现代服务业改革发展举措，服务新业态新模式异军突起，促进了各行业融合升级。深化农业供给侧结构性改革，新型经营主体大批涌现，种植业适度规模经营比重从30%提升到40%以上。采取措施增加中低收入者收入，推动传统消费提档升级、新兴消费快速兴起，网上零售额年均增长30%以上，社会消费品零售总额年均增长11.3%。优化投资结构，鼓励民间投资，发挥政府投资撬动作用，引导更多资金投向强基础、增后劲、惠民生领域。高速铁路运营里程从9000多公里增加到2万5千公里、占世界三分之二，高速公路里程从9.6万公里增加到13.6万公里，新建改建农村公路127万公里，新建民航机场46个，开工重大水利工程122项，完成新一轮农村电网改造，建成全球最大的移动宽带网。五年来，发展新动能迅速壮大，经济增长实现由主要依靠投资、出口拉动转向依靠消费、投资、出口协同拉动，由主要依靠第二产业带动转向依靠三次产业共同带动。这是我们多年想实现而没有实现的重大结构性变革。

推进供给侧结构性改革，必须破除要素市场化配置障碍，降低制度性交易成本。针对长期存在的重审批、轻监管、弱服务问题，我们持续深化“放管服”改革，加快转变政府职能，减少微观管理、直接干预，注重加强宏观调控、市场监管和公共服务。五年来，国务院部门行政审批事项削减44%，非行政许可审批彻底终结，中央政府层面核准的企业投资项目减少90%，行政审批中介服务事项压减74%，职业资格许可和认定大幅减少。中央政府定价项目缩减80%，地方政府定价项目缩减50%以上。全面改革工商登记、注册资本等商事制度，企业开办时间缩短三分之一以上。创新和加强事中事后监管，实行“双随机、一公开”，随机抽取检查人员和检查对象、及时公开查处结果，提高了监管效能和公正性。推行“互联网+政务服务”，实施一站式服务等举措。营商环境持续改善，市场活力明显增强，群众办事更加便利。

（三）坚持创新引领发展，着力激发社会创造力，整体创新能力和效率显著提高。实施创新驱动发展战略，优化创新生态，形成多主体协同、全方位推进的创新局面。扩大科研机构和高校科研自主权，改进科研项目和经费管理，深化科技成果权益管理改革。推进全面创新改革试验，支持北京、上海建设科技创新中心，新设14个国家自主创新示范区，带动形成一批区域创新高地。以企业为主体加强技术创新体系建设，涌现一批具有国际竞争力的创新型企业和新型研发机构。深入开展大众创业、万众创新，实施普惠性支持政策，完善孵化体系。各类市场主体达到9800多万户，五年增加70%以上。国内有效发明专利拥有量增加两倍，技术交易额翻了一番。我国科技创新由跟跑为主转向更多领域并跑、领跑，成为全球瞩目的创新创业热土。

（四）坚持全面深化改革，着力破除体制机制弊端，发展动力不断增强。国资国企改革扎实推进，公司制改革基本完成，兼并重组、压减层级、提质增效取得积极进展。国有企业效益明显好转，去年利润增长23.5%。深化能源、铁路、盐业等领域改革。放宽非公有制经济市场准入。建立不动产统一登记制度。完善产权保护制度。财税改革取得重大进展，全面推行财政预决算公开，构建以共享税为主的中央和地方收入分配格局，启动中央与地方财政事权和支出责任划分改革，中央对地方一般性转移支付规模大幅增加、专项转移支付项目减少三分之二。基本放开利率管制，建立存款保险制度，推动大中型商业银行设立普惠金融事业部，深化政策性、开发性金融机构改革，强化金融监管协调机制。稳步推进教育综合改革，完善城乡义务教育均衡发展促进机制，改革考试招生制度。建立统一的城乡居民基本养老、医疗保险制度，实现机关事业单位和企业养老保险制度并轨。出台划转部分国有资本充实社保基金方案。实施医疗、医保、医药联动改革，全面推开公立医院综合改革，取消长期实行的药品加成政策，药品医疗器械审批制度改革取得突破。推进农村承包地“三权”分置改革、确权面积超过80%，改革重要农产品收储制度。完善主体功能区制度，建立生态文明绩效考评和责任追究制度，推行河长制、湖长制，开展省级以下环保机构垂直管理制度改革试点。各领域改革的深化，推动了经济社会持续健康发展。

（五）坚持对外开放的基本国策，着力实现合作共赢，开放型经济水平显著提升。倡导和推动共建“一带一路”，发起创办亚投行，设立丝路基金，一批重大互联互通、经贸合作项目落地。在上海等省市设立11个自贸试验区，一批改革试点成果向全国推广。改革出口退税负担机制、退税增量全部由中央财政负担，设立13个跨境电商综合试验区，国际贸易“单一窗口”覆盖全国，货物通关时间平均缩短一半以上，进出口实现回稳向好。外商投资由审批制转向负面清单管理，限制性措施削减三分之二。外商投资结构优化，高技术产业占比提高一倍。加大引智力度，来华工作的外国专家增加40%。引导对外投资健康发展。推进国际产能合作，高铁、核电等装备走向世界。新签和升级8个自由贸易协定。沪港通、深港通、债券通相继启动，人民币加入国际货币基金组织特别提款权货币篮子，人民币国际化迈出重要步伐。中国开放的扩大，有力促进了自身发展，给世界带来重大机遇。

（六）坚持实施区域协调发展和新型城镇化战略，着力推动平衡发展，新的增长极增长带加快成长。积极推进京津冀协同发展、长江经济带发展，编制实施相关规划，建设一批重点项目。出台一系列促进西部开发、东北振兴、中部崛起、东部率先发展的改革创新举措。加大对革命老区、民族地区、边疆地区、贫困地区扶持力度，加强援藏援疆援青工作。海洋保护和开发有序推进。实施重点城市群规划，促进大中小城市和小城镇协调发展。绝大多数城市放宽落户限制，居住证制度全面实施，城镇基本公共服务向常住人口覆盖。城乡区域发展协调性显著增强。

（七）坚持以人民为中心的发展思想，着力保障和改善民生，人民群众获得感不断增强。在财力紧张情况下，持续加大民生投入。全面推进精准扶贫、精准脱贫，健全中央统筹、省负总责、市县抓落实的工作机制，中央财政五年投入专项扶贫资金2800多亿元。实施积极的就业政策，重点群体就业得到较好保障。坚持教育优先发展，财政性教育经费占国内生产总值比例持续超过4%。改善农村义务教育薄弱学校办学条件，提高乡村教师待遇，营养改善计划惠及3600多万农村学生。启动世界一流大学和一流学科建设。重点高校专项招收农村和贫困地区学生人数由1万人增加到10万人。加大对各类学校家庭困难学生资助力度，4.3亿人次受益。劳动年龄人口平均受教育年限提高到10.5年。居民基本医保人均财政补助标准由240元提高到450元，大病保险制度基本建立、已有1700多万人次受益，异地就医住院费用实现直接结算，分级诊疗和医联体建设加快推进。持续合理提高退休人员基本养老金。提高低保、优抚等标准，完善社会救助制度，近6000万低保人员和特困群众基本生活得到保障。建立困难和重度残疾人“两项补贴”制度，惠及2100多万人。实施全面两孩政策。强化基层公共文化服务，加快发展文化事业，文化产业年均增长13%以上。全民健身广泛开展，体育健儿勇创佳绩。

（八）坚持人与自然和谐发展，着力治理环境污染，生态文明建设取得明显成效。树立绿水青山就是金山银山理念，以前所未有的决心和力度加强生态环境保护。重拳整治大气污染，重点地区细颗粒物（PM2.5）平均浓度下降30%以上。加强散煤治理，推进重点行业节能减排，71%的煤电机组实现超低排放。优化能源结构，煤炭消费比重下降8.1个百分点，清洁能源消费比重提高6.3个百分点。提高燃油品质，淘汰黄标车和老旧车2000多万辆。加强重点流域海域水污染防治，化肥农药使用量实现零增长。推进重大生态保护和修复工程，扩大退耕还林还草还湿，加强荒漠化、石漠化、水土流失综合治理。开展中央环保督察，严肃查处违法案件，强化追责问责。积极推动《巴黎协定》签署生效，我国在应对全球气候变化中发挥了重要作用。

（九）坚持依法全面履行政府职能，着力加强和创新社会治理，社会保持和谐稳定。提请全国人大常委会制定修订法律95部，制定修订行政法规195部，修改废止一大批部门规章。省、市、县政府部门制定公布权责清单。开展国务院大督查和专项督查，对积极作为、成效突出的给予表彰和政策激励，对不作为的严肃问责。创新城乡基层治理。完善信访工作制度。扩大法律援助范围。促进安全生产领域改革发展，事故总量和重特大事故数量持续下降。改革完善食品药品监管，强化风险全程管控。加强地震、特大洪灾等防灾减灾救灾工作，健全分级负责、相互协同的应急机制，最大程度降低了灾害损失。加强国家安全。健全社会治安防控体系，依法打击各类违法犯罪，有力维护了公共安全。

贯彻落实党中央全面从严治党部署，加强党风廉政建设和反腐败斗争。深入开展党的群众路线教育实践活动、“三严三实”专题教育、“两学一做”学习教育，认真落实党中央八项规定精神，持之以恒纠正“四风”，严格执行国务院“约法三章”。严控新建政府性楼堂馆所和财政供养人员总量，“三公”经费大幅压减。加强行政监察和审计监督。坚决查处和纠正违法违规行为，严厉惩处腐败分子，反腐败斗争压倒性态势已经形成并巩固发展。

各位代表！

过去五年，民族、宗教、侨务等工作创新推进。支持民族地区加快发展，民族团结进步事业取得长足进展。积极引导宗教与社会主义社会相适应。海外侨胞和归侨侨眷在国家现代化建设中作出了独特贡献。

过去五年，在党中央、中央军委领导下，强军兴军开创新局面。制定新形势下军事战略方针，召开古田全军政治工作会议，深入推进政治建军、改革强军、科技兴军、依法治军，人民军队实现政治生态重塑、组织形态重塑、力量体系重塑、作风形象重塑。有效遂行海上维权、反恐维稳、抢险救灾、国际维和、亚丁湾护航、人道主义救援等重大任务。各方配合基本完成裁减军队员额30万任务。军事装备现代化水平显著提升，军民融合深度发展。军政军民紧密团结。人民军队面貌焕然一新，在中国特色强军之路上迈出坚实步伐。

过去五年，港澳台工作取得新进展。“一国两制”实践不断丰富和发展，宪法和基本法权威在港澳进一步彰显，内地与港澳交流合作深入推进，港珠澳大桥全线贯通，香港、澳门保持繁荣稳定。坚持一个中国原则和“九二共识”，加强两岸经济文化交流合作，实现两岸领导人历史性会晤。坚决反对和遏制“台独”分裂势力，有力维护了台海和平稳定。

过去五年，中国特色大国外交全面推进。成功举办首届“一带一路”国际合作高峰论坛、亚太经济合作组织领导人非正式会议、二十国集团领导人杭州峰会、金砖国家领导人厦门会晤等重大主场外交。习近平主席等国家领导人出访多国，出席联合国系列峰会、气候变化大会、世界经济论坛、东亚合作领导人系列会议等重大活动，全方位外交布局深入展开。倡导构建人类命运共同体，为全球治理体系变革贡献更多中国智慧。经济外交、人文交流卓有成效。坚定维护国家主权和海洋权益。中国作为负责任大国，在解决国际和地区热点问题上发挥了重要建设性作用，为世界和平与发展作出新的重大贡献。

各位代表！

回顾过去五年，诸多矛盾交织叠加，各种风险挑战接踵而至，国内外很多情况是改革开放以来没有碰到过的，我国改革发展成就实属来之不易。这是以习近平同志为核心的党中央坚强领导的结果，是习近平新时代中国特色社会主义思想科学指引的结果，是全党全军全国各族人民团结奋斗的结果。我代表国务院，向全国各族人民，向各民主党派、各人民团体和各界人士，表示诚挚感谢！向香港特别行政区同胞、澳门特别行政区同胞、台湾同胞和海外侨胞，表示诚挚感谢！向关心和支持中国现代化建设的各国政府、国际组织和各国朋友，表示诚挚感谢！

安不忘危，兴不忘忧。我们清醒认识到，我国仍处于并将长期处于社会主义初级阶段，仍是世界最大发展中国家，发展不平衡不充分的一些突出问题尚未解决。经济增长内生动力还不够足，创新能力还不够强，发展质量和效益不够高，一些企业特别是中小企业经营困难，民间投资增势疲弱，部分地区经济下行压力较大，金融等领域风险隐患不容忽视。脱贫攻坚任务艰巨，农业农村基础仍然薄弱，城乡区域发展和收入分配差距依然较大。重特大安全生产事故时有发生。在空气质量、环境卫生、食品药品安全和住房、教育、医疗、就业、养老等方面，群众还有不少不满意的地方。政府职能转变还不到位。政府工作存在不足，有些改革举措和政策落实不力，一些干部服务意识和法治意识不强、工作作风不实、担当精神不够，形式主义、官僚主义不同程度存在。群众和企业对办事难、乱收费意见较多。一些领域不正之风和腐败问题仍然多发。我们一定要以对国家和人民高度负责的精神，以不畏艰难的勇气、坚忍不拔的意志，尽心竭力做好工作，使人民政府不负人民重托！

二、2018年经济社会发展总体要求和政策取向

今年是全面贯彻党的十九大精神的开局之年，是改革开放40周年，是决胜全面建成小康社会、实施“十三五”规划承上启下的关键一年。做好政府工作，要在以习近平同志为核心的党中央坚强领导下，以马克思列宁主义、毛泽东思想、邓小平理论、“三个代表”重要思想、科学发展观、习近平新时代中国特色社会主义思想为指导，全面深入贯彻党的十九大和十九届二中、三中全会精神，贯彻党的基本理论、基本路线、基本方略，坚持和加强党的全面领导，坚持稳中求进工作总基调，坚持新发展理念，紧扣我国社会主要矛盾变化，按照高质量发展的要求，统筹推进“五位一体”总体布局和协调推进“四个全面”战略布局，坚持以供给侧结构性改革为主线，统筹推进稳增长、促改革、调结构、惠民生、防风险各项工作，大力推进改革开放，创新和完善宏观调控，推动质量变革、效率变革、动力变革，特别在打好防范化解重大风险、精准脱贫、污染防治的攻坚战方面取得扎实进展，引导和稳定预期，加强和改善民生，促进经济社会持续健康发展。

综合分析国内外形势，我国发展面临的机遇和挑战并存。世界经济有望继续复苏，但不稳定不确定因素很多，主要经济体政策调整及其外溢效应带来变数，保护主义加剧，地缘政治风险上升。我国经济正处在转变发展方式、优化经济结构、转换增长动力的攻关期，还有很多坡要爬、坎要过，需要应对可以预料和难以预料的风险挑战。实践表明，中国的发展成就从来都是在攻坚克难中取得的。当前我国物质技术基础更加雄厚，产业体系完备、市场规模巨大、人力资源丰富、创业创新活跃，综合优势明显，有能力有条件实现更高质量、更有效率、更加公平、更可持续的发展。

今年发展主要预期目标是：国内生产总值增长6.5%左右；居民消费价格涨幅3%左右；城镇新增就业1100万人以上，城镇调查失业率5.5%以内，城镇登记失业率4.5%以内；居民收入增长和经济增长基本同步；进出口稳中向好，国际收支基本平衡；单位国内生产总值能耗下降3%以上，主要污染物排放量继续下降；供给侧结构性改革取得实质性进展，宏观杠杆率保持基本稳定，各类风险有序有效防控。

上述主要预期目标，考虑了决胜全面建成小康社会需要，符合我国经济已由高速增长阶段转向高质量发展阶段实际。从经济基本面和就业吸纳能力看，6.5%左右的增速可以实现比较充分的就业。城镇调查失业率涵盖农民工等城镇常住人口，今年首次把这一指标作为预期目标，以更全面反映就业状况，更好体现共享发展要求。

今年要继续创新和完善宏观调控，把握好宏观调控的度，保持宏观政策连续性稳定性，加强财政、货币、产业、区域等政策协调配合。

积极的财政政策取向不变，要聚力增效。今年赤字率拟按2.6%安排，比去年预算低0.4个百分点；财政赤字2.38万亿元，其中中央财政赤字1.55万亿元，地方财政赤字8300亿元。调低赤字率，主要是我国经济稳中向好、财政增收有基础，也为宏观调控留下更多政策空间。今年全国财政支出21万亿元，支出规模进一步加大。中央对地方一般性转移支付增长10.9%，增强地方特别是中西部地区财力。优化财政支出结构，提高财政支出的公共性、普惠性，加大对三大攻坚战的支持，更多向创新驱动、“三农”、民生等领域倾斜。当前财政状况出现好转，各级政府仍要坚持过紧日子，执守简朴、力戒浮华，严控一般性支出，把宝贵的资金更多用于为发展增添后劲、为民生雪中送炭。

稳健的货币政策保持中性，要松紧适度。管好货币供给总闸门，保持广义货币M2、信贷和社会融资规模合理增长，维护流动性合理稳定，提高直接融资特别是股权融资比重。疏通货币政策传导渠道，用好差别化准备金、差异化信贷等政策，引导资金更多投向小微企业、“三农”和贫困地区，更好服务实体经济。

做好今年工作，要认真贯彻习近平新时代中国特色社会主义经济思想，坚持稳中求进工作总基调，把稳和进作为一个整体来把握，注重以下几点。一是大力推动高质量发展。发展是解决我国一切问题的基础和关键。要着力解决发展不平衡不充分问题，围绕建设现代化经济体系，坚持质量第一、效益优先，促进经济结构优化升级。要尊重经济规律，远近结合，确保经济运行在合理区间，实现经济平稳增长和质量效益提高互促共进。二是加大改革开放力度。改革开放是决定当代中国命运的关键一招，也是实现“两个一百年”奋斗目标的关键一招。在新的历史起点上，思想要再解放，改革要再深化，开放要再扩大。充分发挥人民首创精神，鼓励各地从实际出发，敢闯敢试，敢于碰硬，把改革开放不断向前推进。三是抓好决胜全面建成小康社会三大攻坚战。要分别提出工作思路和具体举措，排出时间表、路线图、优先序，确保风险隐患得到有效控制，确保脱贫攻坚任务全面完成，确保生态环境质量总体改善。我们所做的一切工作，都是为了人民。要坚持以人民为中心的发展思想，从我国基本国情出发，尽力而为、量力而行，把群众最关切最烦心的事一件一件解决好，促进社会公平正义和人的全面发展，使人民生活随着国家发展一年比一年更好。

三、对2018年政府工作的建议

今年经济社会发展任务十分繁重。要紧紧抓住大有可为的历史机遇期，统筹兼顾、突出重点，扎实做好各项工作。

（一）深入推进供给侧结构性改革。坚持把发展经济着力点放在实体经济上，继续抓好“三去一降一补”，大力简政减税减费，不断优化营商环境，进一步激发市场主体活力，提升经济发展质量。

发展壮大新动能。做大做强新兴产业集群，实施大数据发展行动，加强新一代人工智能研发应用，在医疗、养老、教育、文化、体育等多领域推进“互联网+”。加快发展现代服务业。发展智能产业，拓展智能生活，建设智慧社会。运用新技术、新业态、新模式，大力改造提升传统产业。加强新兴产业统计。加大网络提速降费力度，实现高速宽带城乡全覆盖，扩大公共场所免费上网范围，明显降低家庭宽带、企业宽带和专线使用费，取消流量“漫游”费，移动网络流量资费年内至少降低30%，让群众和企业切实受益，为数字中国、网络强国建设加油助力。

加快制造强国建设。推动集成电路、第五代移动通信、飞机发动机、新能源汽车、新材料等产业发展，实施重大短板装备专项工程，推进智能制造，发展工业互联网平台，创建“中国制造2025”示范区。大幅压减工业生产许可证，强化产品质量监管。全面开展质量提升行动，推进与国际先进水平对标达标，弘扬劳模精神和工匠精神，建设知识型、技能型、创新型劳动者大军，来一场中国制造的品质革命。

继续破除无效供给。坚持用市场化法治化手段，严格执行环保、质量、安全等法规标准，化解过剩产能、淘汰落后产能。今年再压减钢铁产能3000万吨左右，退出煤炭产能1.5亿吨左右，淘汰关停不达标的30万千瓦以下煤电机组。加大“僵尸企业”破产清算和重整力度，做好职工安置和债务处置。减少无效供给要抓出新成效。

深化“放管服”改革。全面实施全国统一的市场准入负面清单制度。在全国推开“证照分离”改革，重点是照后减证，各类证能减尽减、能合则合，进一步压缩企业开办时间。大幅缩短商标注册周期。工程建设项目审批时间再压减一半。推进企业投资项目承诺制改革试点。全面实施“双随机、一公开”监管，决不允许假冒伪劣滋生蔓延，决不允许执法者吃拿卡要。深入推进“互联网+政务服务”，使更多事项在网上办理，必须到现场办的也要力争做到“只进一扇门”、“最多跑一次”。加强政务服务标准化建设。大力推进综合执法机构机制改革，着力解决多头多层重复执法问题。加快政府信息系统互联互通，打通信息孤岛。清理群众和企业办事的各类证明，没有法律法规依据的一律取消。优化营商环境就是解放生产力、提高竞争力，要破障碍、去烦苛、筑坦途，为市场主体添活力，为人民群众增便利。

进一步减轻企业税负。改革完善增值税制度，按照三档并两档方向调整税率水平，重点降低制造业、交通运输等行业税率，提高小规模纳税人年销售额标准。大幅扩展享受减半征收所得税优惠政策的小微企业范围。大幅提高企业新购入仪器设备税前扣除上限。实施企业境外所得综合抵免政策。扩大物流企业仓储用地税收优惠范围。继续实施企业重组土地增值税、契税等到期优惠政策。全年再为企业和个人减税8000多亿元，促进实体经济转型升级，着力激发市场活力和社会创造力。

大幅降低企业非税负担。进一步清理规范行政事业性收费，调低部分政府性基金征收标准。继续阶段性降低企业“五险一金”缴费比例。降低电网环节收费和输配电价格，一般工商业电价平均降低10%。深化收费公路制度改革，降低过路过桥费用。加大中介服务收费清理整顿力度。全年要为市场主体减轻非税负担3000多亿元，不合理的坚决取消，过高的坚决降下来，让企业轻装上阵、聚力发展。

（二）加快建设创新型国家。把握世界新一轮科技革命和产业变革大势，深入实施创新驱动发展战略，不断增强经济创新力和竞争力。

加强国家创新体系建设。强化基础研究、应用基础研究和原始创新，启动一批科技创新重大项目，高标准建设国家实验室。鼓励企业牵头实施重大科技项目，支持科研院所、高校与企业融通创新，加快创新成果转化应用。国家科技投入要向民生领域倾斜，加强雾霾治理研究，推进癌症等重大疾病防治攻关，使科技更好造福人民。

落实和完善创新激励政策。改革科技管理制度，科研项目绩效评价要加快从重过程向重结果转变。赋予创新团队和领军人才更大的人财物支配权和技术路线决策权。对承担重大科技攻关任务的科研人员，采取灵活的薪酬制度和奖励措施。探索赋予科研人员科技成果所有权或长期使用权。有悖于激励创新的陈规旧章，要抓紧修改废止；有碍于释放创新活力的繁文缛节，要下决心砍掉。

促进大众创业、万众创新上水平。我国拥有世界上规模最大的人力人才资源，这是创新发展的最大“富矿”。要提供全方位创新创业服务，推进“双创”示范基地建设，鼓励大企业、高校和科研院所等开放创新资源，发展平台经济、共享经济，形成线上线下结合、产学研用协同、大中小企业融合的创新创业格局，打造“双创”升级版。设立国家融资担保基金，支持优质创新型企业上市融资，将创业投资、天使投资税收优惠政策试点范围扩大到全国。深化人才发展体制改革，推动人力资源自由有序流动，支持企业提高技术工人待遇，加大高技能人才激励，鼓励海外留学人员回国创新创业，拓宽外国人才来华绿色通道。集众智汇众力，一定能跑出中国创新“加速度”。

（三）深化基础性关键领域改革。以改革开放40周年为重要契机，推动改革取得新突破，不断解放和发展社会生产力。

推进国资国企改革。制定出资人监管权责清单。深化国有资本投资、运营公司等改革试点，赋予更多自主权。继续推进国有企业优化重组和央企股份制改革，加快形成有效制衡的法人治理结构和灵活高效的市场化经营机制，持续瘦身健体，提升主业核心竞争力，推动国有资本做强做优做大。积极稳妥推进混合所有制改革。落实向全国人大常委会报告国有资产管理情况的制度。国有企业要通过改革创新，走在高质量发展前列。

支持民营企业发展。坚持“两个毫不动摇”，坚持权利平等、机会平等、规则平等，全面落实支持非公有制经济发展的政策措施，认真解决民营企业反映的突出问题，坚决破除各种隐性壁垒。构建亲清新型政商关系，健全企业家参与涉企政策制定机制。激发和保护企业家精神，壮大企业家队伍，增强企业家信心，让民营企业在市场经济浪潮中尽显身手。

完善产权制度和要素市场化配置机制。产权制度是社会主义市场经济的基石。要以保护产权、维护契约、统一市场、平等交换、公平竞争为基本导向，完善相关法律法规。对各种侵权行为要依法严肃处理，对产权纠纷申诉案件要依法甄别纠正。强化知识产权保护，实行侵权惩罚性赔偿制度。加快技术、土地等要素价格市场化改革，深化资源类产品和公共服务价格改革，打破行政垄断，防止市场垄断。要用有力的产权保护、顺畅的要素流动，让市场活力和社会创造力竞相迸发。

深化财税体制改革。推进中央与地方财政事权和支出责任划分改革，抓紧制定收入划分改革方案，完善转移支付制度。健全地方税体系，稳妥推进房地产税立法。改革个人所得税。全面实施绩效管理，使财政资金花得其所、用得安全。

加快金融体制改革。改革完善金融服务体系，支持金融机构扩展普惠金融业务，规范发展地方性中小金融机构，着力解决小微企业融资难、融资贵问题。深化多层次资本市场改革，推动债券、期货市场发展。拓展保险市场的风险保障功能。改革金融监管体制。深化利率汇率市场化改革，保持人民币汇率在合理均衡水平上的基本稳定。

推进社会体制改革。深化养老保险制度改革，建立企业职工基本养老保险基金中央调剂制度。深化公立医院综合改革，协调推进医疗价格、人事薪酬、药品流通、医保支付改革，提高医疗卫生服务质量，下大力气解决群众看病就医难题。深入推进教育、文化、体育等改革，充分释放社会领域巨大发展潜力。

健全生态文明体制。改革完善生态环境管理制度，加强自然生态空间用途管制，推行生态环境损害赔偿制度，完善生态补偿机制，以更加有效的制度保护生态环境。

（四）坚决打好三大攻坚战。要围绕完成年度攻坚任务，明确各方责任，强化政策保障，把各项工作做实做好。

推动重大风险防范化解取得明显进展。当前我国经济金融风险总体可控，要标本兼治，有效消除风险隐患。严厉打击非法集资、金融诈骗等违法犯罪活动。加快市场化法治化债转股和企业兼并重组。加强金融机构风险内控。强化金融监管统筹协调，健全对影子银行、互联网金融、金融控股公司等监管，进一步完善金融监管、提升监管效能。防范化解地方政府债务风险。严禁各类违法违规举债、担保等行为。省级政府对本辖区债务负总责，省级以下各级地方政府各负其责，积极稳妥处置存量债务。健全规范的地方政府举债融资机制。今年安排地方专项债券1.35万亿元，比去年增加5500亿元，优先支持在建项目平稳建设，合理扩大专项债券使用范围。我国经济基本面好，政策工具多，完全能够守住不发生系统性风险的底线。

加大精准脱贫力度。今年再减少农村贫困人口1000万以上，完成易地扶贫搬迁280万人。深入推进产业、教育、健康、生态和文化等扶贫，补齐基础设施和公共服务短板，加强东西部扶贫协作和对口支援，注重扶贫同扶志、扶智相结合，激发脱贫内生动力。强化对深度贫困地区支持，中央财政新增扶贫投入及有关转移支付向深度贫困地区倾斜。对老年人、残疾人、重病患者等特定贫困人口，因户因人落实保障措施。攻坚期内脱贫不脱政策，新产生的贫困人口和返贫人口要及时纳入帮扶。加强扶贫资金整合和绩效管理。开展扶贫领域腐败和作风问题专项治理，改进考核监督方式。坚持现行脱贫标准，确保进度和质量，让脱贫得到群众认可、经得起历史检验。

推进污染防治取得更大成效。巩固蓝天保卫战成果，今年二氧化硫、氮氧化物排放量要下降3%，重点地区细颗粒物（PM2.5）浓度继续下降。推动钢铁等行业超低排放改造。提高污染排放标准，实行限期达标。大力发展清洁能源。开展柴油货车、船舶超标排放专项治理，继续淘汰老旧车。深入推进水、土壤污染防治，今年化学需氧量、氨氮排放量要下降2%。实施重点流域和海域综合治理，全面整治黑臭水体。加大污水处理设施建设力度，完善收费政策。加强固体废弃物和垃圾分类处置，严禁“洋垃圾”入境。加强生态系统保护和修复，全面划定并严守生态保护红线，完成造林1亿亩以上，耕地轮作休耕试点面积增加到3000万亩，加强地下水保护和修复，扩大湿地保护和恢复范围，深化国家公园体制改革试点。严控填海造地。严格环境执法和问责。我们要携手行动，建设天蓝、地绿、水清的美丽中国。

（五）大力实施乡村振兴战略。科学制定规划，健全城乡融合发展体制机制，依靠改革创新壮大乡村发展新动能。

推进农业供给侧结构性改革。促进农林牧渔业和种业创新发展，加快建设现代农业产业园和特色农产品优势区。 坚持提质导向，稳定和优化粮食生产。加快消化粮食库存。发展农产品加工业。新增高标准农田8000万亩以上、高效节水灌溉面积2000万亩。培育新型经营主体，提高农业科技水平，推进农业机械化全程全面发展，加强面向小农户的社会化服务。鼓励支持返乡农民工、大中专毕业生、科技人员、退役军人和工商企业等从事现代农业建设、发展农村新业态新模式。深入推进“互联网+农业”，多渠道增加农民收入，促进农村一二三产业融合发展。

全面深化农村改革。落实第二轮土地承包到期后再延长30年的政策。探索宅基地所有权、资格权、使用权分置改革。改进耕地占补平衡管理办法，建立新增耕地指标、城乡建设用地增减挂钩节余指标跨省域调剂机制，所得收益全部用于脱贫攻坚和支持乡村振兴。深化粮食收储、集体产权、集体林权、国有林区林场、农垦、供销社等改革，使农业农村充满生机活力。

推动农村各项事业全面发展。完善农村医疗、教育、文化等公共服务。改善供水、供电、信息等基础设施，新建改建农村公路20万公里。稳步开展农村人居环境整治三年行动，推进“厕所革命”和垃圾收集处理。促进农村移风易俗。健全自治、法治、德治相结合的乡村治理体系。大力培育乡村振兴人才。我们要坚持走中国特色社会主义乡村振兴道路，加快实现农业农村现代化。

（六）扎实推进区域协调发展战略。完善区域发展政策，推进基本公共服务均等化，逐步缩小城乡区域发展差距，把各地比较优势和潜力充分发挥出来。

塑造区域发展新格局。加强对革命老区、民族地区、边疆地区、贫困地区改革发展的支持。以疏解北京非首都功能为重点推进京津冀协同发展，高起点规划、高标准建设雄安新区。以生态优先、绿色发展为引领推进长江经济带发展。出台实施粤港澳大湾区发展规划纲要，全面推进内地同香港、澳门互利合作。制定西部大开发新的指导意见，落实东北等老工业基地振兴举措，继续推动中部地区崛起，支持东部地区率先发展。加强对资源型地区经济转型发展的支持。壮大海洋经济，坚决维护国家海洋权益。

提高新型城镇化质量。今年再进城落户1300万人，加快农业转移人口市民化。完善城镇规划，优先发展公共交通，健全菜市场、停车场等便民服务设施，加快无障碍设施建设。有序推进“城中村”、老旧小区改造，完善配套设施，鼓励有条件的加装电梯。加强排涝管网、地下综合管廊、海绵城市等建设。新型城镇化的核心在人，要加强精细化服务、人性化管理，使人人都有公平发展机会，让居民生活得方便、舒心。

（七）积极扩大消费和促进有效投资。顺应居民需求新变化扩大消费，着眼调结构增加投资，形成供给结构优化和总需求适度扩大的良性循环。

增强消费对经济发展的基础性作用。推进消费升级，发展消费新业态新模式。将新能源汽车车辆购置税优惠政策再延长三年，全面取消二手车限迁政策。支持社会力量增加医疗、养老、教育、文化、体育等服务供给。创建全域旅游示范区，降低重点国有景区门票价格。推动网购、快递健康发展。对各类侵害消费者权益的行为，要依法惩处、决不姑息。

发挥投资对优化供给结构的关键性作用。今年要完成铁路投资7320亿元、公路水运投资1.8万亿元左右，水利在建投资规模达到1万亿元。重大基础设施建设继续向中西部地区倾斜。实施新一轮重大技术改造升级工程。中央预算内投资安排5376亿元，比去年增加300亿元。落实鼓励民间投资政策措施，在铁路、民航、油气、电信等领域推出一批有吸引力的项目，务必使民间资本进得来、能发展。

（八）推动形成全面开放新格局。进一步拓展开放范围和层次，完善开放结构布局和体制机制，以高水平开放推动高质量发展。

推进“一带一路”国际合作。坚持共商共建共享，落实“一带一路”国际合作高峰论坛成果。推动国际大通道建设，深化沿线大通关合作。扩大国际产能合作，带动中国制造和中国服务走出去。优化对外投资结构。加大西部、内陆和沿边开放力度，提高边境跨境经济合作区发展水平，拓展开放合作新空间。

促进外商投资稳定增长。加强与国际通行经贸规则对接，建设国际一流营商环境。全面放开一般制造业，扩大电信、医疗、教育、养老、新能源汽车等领域开放。有序开放银行卡清算等市场，放开外资保险经纪公司经营范围限制，放宽或取消银行、证券、基金管理、期货、金融资产管理公司等外资股比限制，统一中外资银行市场准入标准。实施境外投资者境内利润再投资递延纳税。简化外资企业设立程序，商务备案与工商登记“一口办理”。全面复制推广自贸试验区经验，探索建设自由贸易港，打造改革开放新高地。

巩固外贸稳中向好势头。扩大出口信用保险覆盖面，整体通关时间再压缩三分之一。改革服务贸易发展机制。培育贸易新业态新模式。推动加工贸易向中西部梯度转移。积极扩大进口，办好首届中国国际进口博览会，下调汽车、部分日用消费品等进口关税。我们要以更大力度的市场开放，促进产业升级和贸易平衡发展，为消费者提供更多选择。

促进贸易和投资自由化便利化。中国坚定不移推进经济全球化，维护自由贸易，愿同有关方推动多边贸易谈判进程，早日结束区域全面经济伙伴关系协定谈判，加快亚太自贸区和东亚经济共同体建设。中国主张通过平等协商解决贸易争端，反对贸易保护主义，坚决捍卫自身合法权益。

（九）提高保障和改善民生水平。要在发展基础上多办利民实事、多解民生难事，兜牢民生底线，不断提升人民群众的获得感、幸福感、安全感。

着力促进就业创业。加强全方位公共就业服务，大规模开展职业技能培训，运用“互联网+”发展新就业形态。今年高校毕业生820多万人，再创历史新高，要促进多渠道就业，支持以创业带动就业。扎实做好退役军人安置、管理和保障工作。加大对残疾人等就业困难人员援助力度。扩大农民工就业，全面治理拖欠工资问题。要健全劳动关系协商机制，消除性别和身份歧视，使更加公平、更加充分的就业成为我国发展的突出亮点。

稳步提高居民收入水平。继续提高退休人员基本养老金和城乡居民基础养老金。合理调整社会最低工资标准。完善机关事业单位工资和津补贴制度，向艰苦地区、特殊岗位倾斜。提高个人所得税起征点，增加子女教育、大病医疗等专项费用扣除，合理减负，鼓励人民群众通过劳动增加收入、迈向富裕。

发展公平而有质量的教育。推动城乡义务教育一体化发展，教育投入继续向困难地区和薄弱环节倾斜。切实降低农村学生辍学率，抓紧消除城镇“大班额”，着力解决中小学生课外负担重问题。儿童是民族的未来、家庭的希望。要多渠道增加学前教育资源供给，重视对幼儿教师的关心和培养，运用互联网等信息化手段对儿童托育中育儿过程加强监管，一定要让家长放心安心。大力发展职业教育，支持和规范社会力量举办职业教育。推进普及高中阶段教育。以经济社会发展需要为导向，优化高等教育结构，加快“双一流”建设，支持中西部建设有特色、高水平大学。继续实施农村和贫困地区专项招生计划。发展民族教育、特殊教育、继续教育和网络教育。加强师资队伍和师德师风建设。要加快推进教育现代化，办好人民满意的教育，让每个人都有平等机会通过教育改变自身命运、成就人生梦想。

实施健康中国战略。提高基本医保和大病保险保障水平，居民基本医保人均财政补助标准再增加40元，一半用于大病保险。扩大跨省异地就医直接结算范围，把基层医院和外出农民工、外来就业创业人员等全部纳入。加大医护人员培养力度，加强全科医生、儿科医生队伍建设，推进分级诊疗和家庭医生签约服务。继续提高基本公共卫生服务经费人均财政补助标准。坚持预防为主，加强重大疾病防控。改善妇幼保健服务。支持中医药事业传承创新发展。鼓励中西医结合。创新食品药品监管方式，注重用互联网、大数据等提升监管效能，加快实现全程留痕、信息可追溯，让问题产品无处藏身、不法制售者难逃法网，让消费者买得放心、吃得安全。做好北京冬奥会、冬残奥会筹办工作，多渠道增加全民健身场所和设施。人民群众身心健康、向善向上，国家必将生机勃勃、走向繁荣富强。

更好解决群众住房问题。启动新的三年棚改攻坚计划，今年开工580万套。加大公租房保障力度，对低收入住房困难家庭要应保尽保，将符合条件的新就业无房职工、外来务工人员纳入保障范围。坚持房子是用来住的、不是用来炒的定位，落实地方主体责任，继续实行差别化调控，建立健全长效机制，促进房地产市场平稳健康发展。支持居民自住购房需求，培育住房租赁市场，发展共有产权住房。加快建立多主体供给、多渠道保障、租购并举的住房制度，让广大人民群众早日实现安居宜居。

强化民生兜底保障。稳步提高城乡低保、社会救助、抚恤优待等标准。积极应对人口老龄化，发展居家、社区和互助式养老，推进医养结合，提高养老院服务质量。关心帮助农村留守儿童。加强城乡困境儿童保障。做好伤残军人和军烈属优抚工作。加强残疾人康复服务。健全社会救助体系，支持公益慈善事业发展。倾情倾力做好托底工作，不因事难而推诿，不因善小而不为，要让每一个身处困境者都能得到社会的关爱和温暖。

打造共建共治共享社会治理格局。完善基层群众自治制度，加强社区治理。发挥好工会、共青团、妇联等群团组织作用。促进社会组织、专业社会工作、志愿服务健康发展。加强社会信用体系建设。完善公共法律服务体系，落实普法责任制。营造尊重妇女、关爱儿童、尊敬老人、爱护残疾人的良好风尚。完善社会矛盾纠纷多元化解机制。创新信访工作方式，依法及时解决群众合理诉求。严格落实安全生产责任，坚决遏制重特大事故。做好地震、气象、地质等工作，提高防灾减灾救灾能力。推进平安中国建设，严密防范和坚决打击暴力恐怖活动，依法开展扫黑除恶专项斗争，惩治盗抢骗黄赌毒等违法犯罪活动，整治电信网络诈骗、侵犯公民个人信息、网络传销等突出问题，维护国家安全和公共安全。

为人民过上美好生活提供丰富精神食粮。要弘扬中华优秀传统文化，继承革命文化，发展社会主义先进文化，培育和践行社会主义核心价值观。加强思想道德建设和群众性精神文明创建。加快构建中国特色哲学社会科学，繁荣文艺创作，发展新闻出版、广播影视、档案等事业。加强文物保护利用和文化遗产保护传承。建好新型智库。加强互联网内容建设。深入实施文化惠民工程，培育新型文化业态，加快文化产业发展。倡导全民阅读，建设学习型社会。深化中外人文交流，增强中华文化影响力。我们要以中国特色社会主义文化的繁荣兴盛，凝聚起实现民族复兴的磅礴精神力量。

各位代表！

进入新时代，政府工作在新的一年要有新气象新作为。要牢固树立“四个意识”，坚定“四个自信”，坚决维护习近平总书记核心地位，坚决维护党中央权威和集中统一领导，落实全面从严治党要求，加强政府自身建设，深入推进政府职能转变，全面提升履职水平，为人民提供优质高效服务。

全面推进依宪施政、依法行政。严格遵守宪法法律，加快建设法治政府，把政府活动全面纳入法治轨道。坚持严格规范公正文明执法，有权不可任性，用权必受监督。各级政府要依法接受同级人大及其常委会的监督，自觉接受人民政协的民主监督，主动接受社会和舆论监督，认真听取人大代表、政协委员意见，听取民主党派、工商联、无党派人士和各人民团体意见。政府要信守承诺，决不能“新官不理旧账”。全面推进政务公开。推行政府法律顾问制度。坚持科学、民主、依法决策，凡涉及公众利益的重大事项，都要深入听取各方意见包括批评意见。人民政府的所有工作都要体现人民意愿，干得好不好要看实际效果、最终由人民来评判。

全面加强党风廉政建设。推进“两学一做”学习教育常态化制度化，认真开展“不忘初心、牢记使命”主题教育。坚决贯彻落实党中央八项规定及实施细则精神，驰而不息整治“四风”，特别是力戒形式主义、官僚主义。加强审计监督。巩固发展反腐败斗争压倒性态势，把权力关进制度的笼子，坚决惩治各类腐败行为。政府工作人员要廉洁修身，勤勉尽责，自觉接受法律监督、监察监督和人民的监督，干干净净为人民做事，决不辜负人民公仆的称号。

全面提高政府效能。优化政府机构设置和职能配置，深化机构改革，形成职责明确、依法行政的政府治理体系，增强政府公信力和执行力。中国改革发展的一切成就，都是干出来的。对各级政府及其工作人员来说，为人民干事是天职、不干是失职。要完善激励约束、容错纠错机制，旗帜鲜明给积极干事者撑腰鼓劲，对庸政懒政者严肃问责。决不能表态多调门高、行动少落实差，决不允许占着位子不干事。广大干部要提高政治素质和工作本领，求真务实，干字当头，干出实打实的新业绩，干出群众的好口碑，干出千帆竞发、百舸争流的生动局面。

各位代表！

中华民族是一个和睦相处、团结温暖的大家庭。我们要坚持和完善民族区域自治制度，全面贯彻党的民族政策。继续加强对民族地区、人口较少民族发展的支持。组织好广西壮族自治区、宁夏回族自治区成立60周年庆祝活动。加强各民族交往交流交融，让中华民族共同体的根基更加坚实、纽带更加牢固。

我们要全面贯彻党的宗教工作基本方针，坚持我国宗教的中国化方向，促进宗教关系健康和谐，发挥宗教界人士和信教群众在促进经济社会发展中的积极作用。

我们要认真落实侨务政策，维护海外侨胞和归侨侨眷合法权益，为他们在国家现代化建设中发挥独特优势和重要作用创造更好条件，激励海内外中华儿女同心奋斗、共创辉煌。

各位代表！

面对国家安全环境的深刻变化，我们要以党在新时代的强军目标为引领，牢固确立习近平强军思想在国防和军队建设中的指导地位，坚定不移走中国特色强军之路，全面推进练兵备战工作，坚决有力维护国家主权、安全、发展利益。坚持党对军队绝对领导的根本原则和制度，全面深入贯彻军委主席负责制。继续推进国防和军队改革，建设强大稳固的现代边海空防。完善国防动员体系，加强全民国防教育。维护军人军属合法权益，让军人成为全社会尊崇的职业。深入实施军民融合发展战略，深化国防科技工业改革。各级政府要采取更有力的举措，支持国防和军队建设改革，深入开展拥军优属、拥政爱民活动，使军政军民团结始终坚如磐石、始终根深叶茂。

各位代表！

我们要继续全面准确贯彻“一国两制”方针，严格依照宪法和基本法办事。全力支持香港、澳门特别行政区政府和行政长官依法施政，大力发展经济、持续改善民生、有序推进民主、促进社会和谐。支持香港、澳门融入国家发展大局，深化内地与港澳地区交流合作。我们坚信，香港、澳门一定能与祖国内地同发展、共繁荣。

我们要继续贯彻对台工作大政方针，坚持一个中国原则，在“九二共识”基础上推动两岸关系和平发展，推进祖国和平统一进程。坚决维护国家主权和领土完整，绝不容忍任何“台独”分裂图谋和行径。扩大两岸经济文化交流合作，逐步为台湾同胞在大陆学习、创业、就业、生活提供与大陆同胞同等待遇。两岸同根，骨肉相亲。两岸同胞顺应历史大势、共担民族大义，必将共创中华民族伟大复兴的美好未来！

各位代表！

中国与世界各国发展紧密相连、命运休戚与共。我们将始终不渝走和平发展道路，推动构建新型国际关系。积极参与改革完善全球治理，致力于建设开放型世界经济。推进大国协调合作，深化同周边国家睦邻友好和共同发展，加强同发展中国家团结合作。办好博鳌亚洲论坛年会、上海合作组织峰会、中非合作论坛峰会等主场外交。继续为解决国际和地区热点问题发挥负责任大国作用。加强和完善海外利益安全保障体系。中国愿与各国一道，为推动构建人类命运共同体不懈努力！

各位代表！

团结凝聚力量，实干创造未来。我们要更加紧密地团结在以习近平同志为核心的党中央周围，高举中国特色社会主义伟大旗帜，以习近平新时代中国特色社会主义思想为指导，锐意进取，扎实工作，促进经济社会持续健康发展，为决胜全面建成小康社会、夺取新时代中国特色社会主义伟大胜利，为把我国建设成为富强民主文明和谐美丽的社会主义现代化强国、实现中华民族伟大复兴的中国梦作出新的贡献！
